# Sixty years of observations and future projections of nine declining North American glaciers

**DOI:** 10.1038/s41598-026-41235-6

**Published:** 2026-03-17

**Authors:** Edward G. Josberger, Robert A. Shuchman, Ray H. Watkins

**Affiliations:** 1 Michigan Tech Research Institute, Michigan Technological University, Ann Arbor, MI 48105 USA; 2 Retired, Washington Water Science Center, U.S. Geological Survey, 1201 Pacific Avenue, Suite 600, Tacoma, 98402 WA USA; 3 Geological and Mining Engineering and Sciences, Michigan Technological University, Houghton, MI 49931 USA

**Keywords:** Applied physics, Cryospheric science

## Abstract

Warming climate has greatly affected the stability of the world’s glaciers. In 1957, as part of the International Geophysical Year, nine North American glaciers were surveyed in great detail so that they could serve as future benchmarks. Since their initial survey, these nine benchmark glaciers have drastically decreased in volume and areal extent. This is a direct result of global warming. However, the exact amount of their change has never been fully documented and predictions of their lifespans in the wake of continued temperature increase has never been projected. In this study, we utilized advanced satellite techniques to quantify changes in the nine glaciers as well as assessed their long-term survivability. The methodology successfully used in this study can be applied to glaciers throughout the world, which would provide a cost-effective evaluation of past, present, and future glacial change at localized scales. We show that since 1957, the glaciers have lost 1.4 metric gigatons (Gt) of freshwater and have shrunk on average by 33%. Additionally, utilizing future temperature projections and cumulatively integrating temperature-driven volume changes over time, we show that only 3 of the 9 glaciers will still be in existence by 2100. These results paint a grim picture for the future generations observing glaciers in North America.

**Supplementary Information:**

The online version contains supplementary material available at 10.1038/s41598-026-41235-6.

## Introduction

Glaciers have existed at least periodically on the surface of the Earth for at least 2 billion years, since before the time of the dinosaurs^1^. However, in the face of record dramatic global temperature rise they too are becoming an endangered species^2,3^. In 1958, during the International Geophysical Year, a suite of federal agencies (DoD, USGS, NPS, and others) coordinated by the American Geophysical Society (AGS) had the foresight to perform a detailed baseline mapping of nine representative glaciers across Alaska and Washington State^4^. Because this dataset is representative of many types of glaciers, the behavior of these glaciers should correlate with the behavior of many North American glaciers. The original 1957/58 survey took over two years to complete and included the use of US Navy aircraft collecting aerial photography as well as extensive on-site glacier surveying. The AGS hoped that these nine glaciers would be periodically resurveyed to ascertain future glacier changes. In 1994, the Geophysical Institute at the University of Alaska, Fairbanks resurveyed the nine glaciers again using aircraft and ground based measurement techniques^5^.

With the development of sophisticated satellite-based remote sensing techniques, we were able to remap these nine glaciers in 2007/08 and 2017/18, thus creating a 60-year data record. The new mapping techniques relied on Digital Elevation Models (DEMs), which are 3D surface representations derived primarily from high-resolution electro-optical satellite data. These DEMs provide both elevation and photographic information about Earth’s surface, enabling detailed analysis of glacial terrain and change (See Methods)^6–8^. Satellite-derived DEMs were used to map changes in surface elevation, which allowed us to estimate glacier thickness, volume, and area change over time. DEMs from ArcticDEM^8^ strip products were used for the 2017/18 period, with the exception of Blue Glacier where USGS high-resolution mosaiced 3DEP DEM was used^9^. For the 2007/08 period, elevation data were obtained from a manually digitized DEMs from orthorectified satellite imagery.

The high-resolution DEM data across all sites enabled additional geospatial analyses, including area change mapping, solar exposure estimation, and land cover classification surrounding each glacier. These satellite-based methods provide a cost-effective, high-quality alternative to time-intensive field campaigns and allow for repeat monitoring on a regional scale. We digitized the original 1957/58 field-generated topographic maps to serve as a consistent historical baseline and merged these with the 2007/08 and 2017/18 elevation observations, as well as the volume and area change estimates from the 1994 survey^5^.

Here, we first describe the nine glaciers and evaluate their elevation change between 1957/58 and 2017/18. We then calculate mass loss rates, volume and area loss, and associated freshwater discharge using the digitized 1958 baseline. The area and volume estimates were found to be robust for most glaciers and time periods, although some limitations apply to specific datasets as noted. Finally, we explore projections for glacier survival under low, medium, and high warming climate scenarios. Alarmingly, most glaciers are projected to disappear by 2100 under the high warming scenario.

## Results

### Nine North American glaciers surveyed

**Fig. 1:**
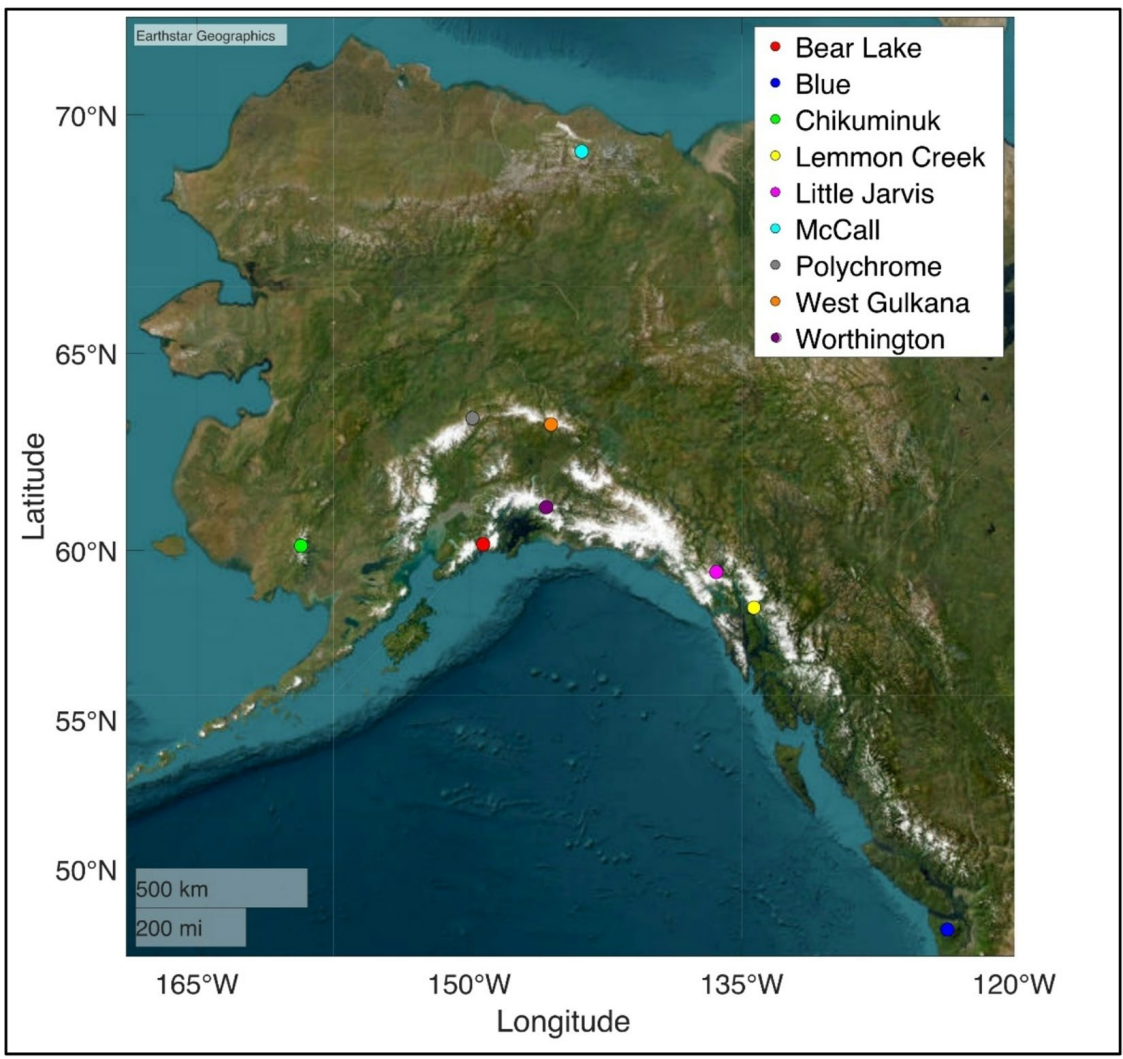
A mapping of the Nine North American Glaciers resurveyed in this study. These glaciers in this study span from the Olympic Peninsula in Washington State to the North Slope of Alaska. These glaciers are largely representative of the various types of glaciers found throughout North America. The nine glaciers include tidewater, valley, polythermal valley, and mountain. Additionally, these nine glaciers have been subject to varying amounts of anthropogenically forced temperature rise. *Basemap generated using MATLAB R2022b Mapping Toolbox with Esri satellite imagery.* Software: MATLAB R2022b, Source: Esri, Maxar, Earthstar Geographics, and the GIS User Community, URL: https://www.mathworks.com/help/map/geobasemap.html.

In this study, we remapped the nine glaciers originally surveyed as part of the IGY in 1958. The glaciers span the Olympic Peninsula in Washington State to the North Slope of Alaska and largely represent the various types of glaciers found throughout North America (Fig. 1). Note from the figure the proximity of each glacier to the ocean. Table 1 summarizes the characteristics of each of the nine glaciers. The table provides location, average elevation, type of glacier, CMIP6-derived temperature range^10–12^, PRISM annual precipitation^13^, and solar exposure — all of which influence mass loss rates and the ultimate survival of the glaciers. The temperature values reflect the annual mean CMIP6 GFDL historical climate at each glacier’s location and are included for contextual comparison only. Southern solar exposure^14^, warmer summer temperatures^15^, proximity to the ocean^16^, and altitude^17^ all have a major influence on the glaciers’ mass loss rates.

**Table 1:** Characteristics of the Nine North American Glaciers. Presented in the table are the unique characteristics of the glaciers, including: Location, Elevation, Glacier Type, CMIP 6 Temperature Range, Proximity to the ocean, Surrounding Terrain Type, Solar exposure, and PRISM Precipitation. Note all but Blue Glacier are in Alaska which is in the Olympic Peninsula in Washington State.

Glacier name	Location	Average elevation (m)	Glacier type	Temp. range (^o^C)	Ocean proximity (km)	Terrain type	Solar exposure	Precipitation (cm/yr)
Bear lake	Kana Peninsula 60^o^N, 149^o^W	750	Valley	−2 to 10	> 50	Vegetated	Northwest	37
Blue	Olympic Peninsula 47^o^N, 124^o^W	1209	Valley	−3 to 15	< 50	Vegetated	North	540
Chikuminuk	Dillingham 60^o^N, 159^o^W	678	Mountain	−10 to 10	> 50	Vegetated	North	51
Lemon creek	Haines 58^o^N, 134^o^W	1066	Valley	−5 to 9	< 50	Vegetated	North	169
Little jarvis	Skagway 59^o^N, 136^o^W	1059	Valley	−6 to 9	< 50	Vegetated	North	135
McCall	North Slope 69^o^N, 144^o^W	1311	Polytherm-al Valley	−25 to 7	> 50	Rock	North West	37
Polychrome	Yukon-Kowukuk 63^o^N, 150^o^W	764	Valley	−13 to 9	> 50	Rock	North	42
West Gulkana	Denali 63^o^N, 146^o^W	1488	Valley	−14 to 8	> 50	Rock	Southern	46
Worthington	Valdez-Cordova 61^o^N, 146^o^W	493	Valley	−6 to 8.5	**>** 50	Vegetated	Eastern	44

**Fig. 2:**
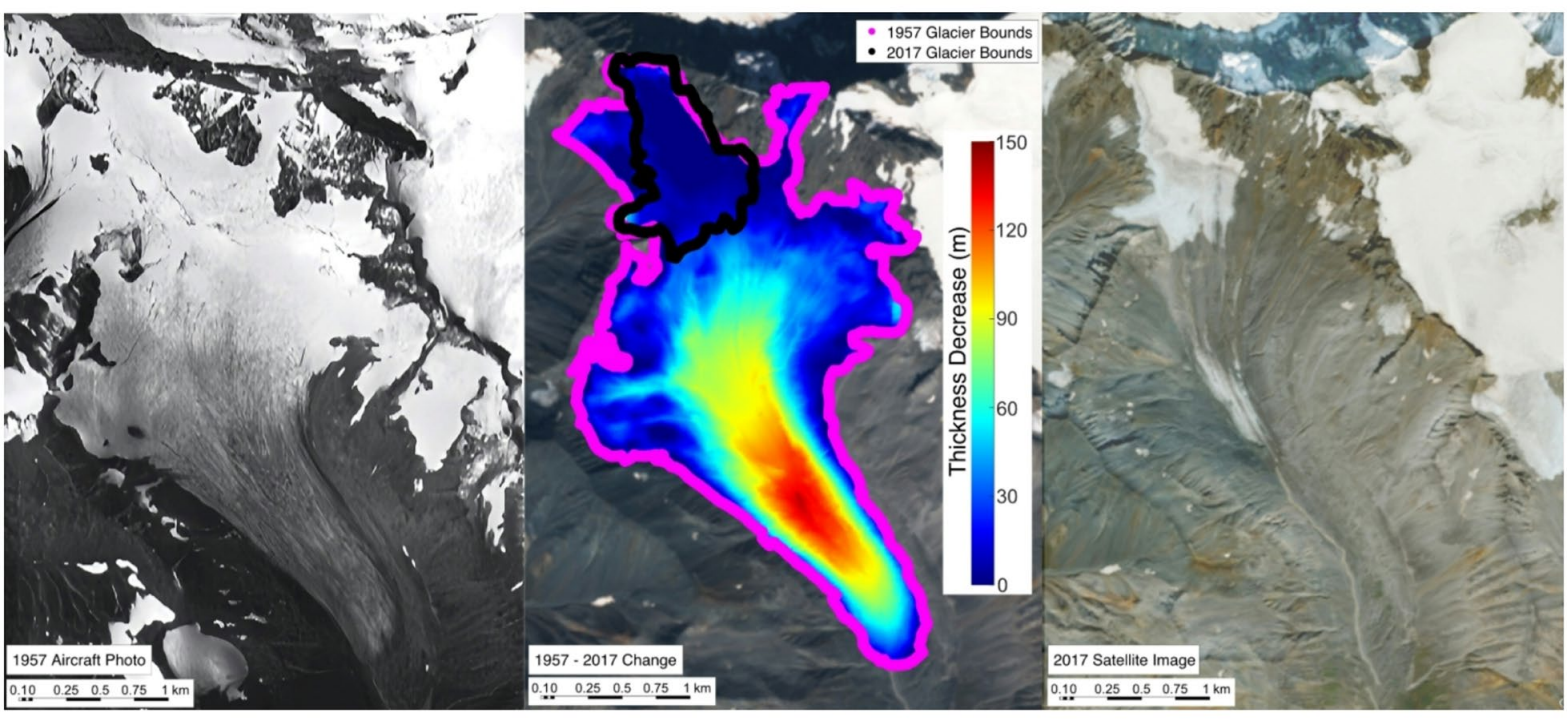
Climate Induced Near-Death of West Gulkana Glacier in Alaska: On the left is a an arial photo of the glacier in 1957, which is in contrast to the 2017 satellite image on the right. During this 60 plus year observation period, West Gulkana Glacier has decreased in volume by approximately 67% and its area has decreased significantly 87% (see center image). Note the terminus has also receded approximately ~3 km. The lost water from the glacial ecosystem equates to 0.15 Gigatons of liquid freshwater. *Left image reproduced from: Seligman*,* G. (1960). Nine Glacier Maps: Northwestern North America. American Geographical Society*,* Special Publication No. 34. Public domain image captured by the U.S. Navy. Right image sourced from Copernicus Sentinel-2*,* under the open data license.* Software: MATLAB R2022b, Satellite Image Source: Copernicus Open Access Hub (Sentinel-2), URL: https://dataspace.copernicus.eu/explore-data.

We demonstrate (Fig. 2) the use of remote sensing technologies to perform global glacier long-term monitoring. The West Gulkana Glacier, photographed by a Navy aircraft in 1957, is shown on the left while the right image shows the glacier in 2017, courtesy of a high-resolution Sentinel-2 satellite image^18^. The center insert is a thickness change map over the time period using DEM technology (see Methods) and shows the near demise of the glacier. During this 60-plus year observation period, West Gulkana Glacier has decreased in volume approximately 67%, and its area has also decreased significantly by ~ 87%. As indicated in the center image (Fig. 2), the glacier terminus has receded ~ 3 km. The glacier decreased in thickness approximately 38 m, resulting in 0.14 metric Gt (See Methods) of liquid freshwater (around 60,000 Olympic-sized swimming pools) permanently removed from the glacier ecosystem.

It is important to note that our glacier volume estimates are based on surface elevation change computed over a fixed glacier extent— the 1957/58 outline—and are not directly dependent on time-varying area measurements. As such, any potential errors in the 2017/18 area delineation, including omission of stagnant debris-covered ice, do not influence our volume change calculations. When surface elevation reaches bedrock, the corresponding pixel’s volume contribution becomes zero, regardless of whether it falls within the modern glacier boundary. This ensures that inconsistencies in late-stage area delineation, particularly for stagnant or disconnected ice, do not propagate into our volume or projection results. In the case of West Gulkana Glacier, the excluded debris-covered portion is interpreted as stagnant, disconnected ice lacking accumulation input or dynamic flow—features characteristic of glacier disconnection^19^. This discontinuity is visually evident in Fig. 2, where the active upper glacier is clearly separated from the lower debris-covered region.

### Glacial changes 1957/58 to 2017/18

To begin, we used the 1957/58 survey data as a baseline for each of the nine glaciers, corrected using glacier-specific vertical offsets as outlined in the 1994 study^5^. We then used published results from the 1994 resurvey, along with our own DEM-based observations from 2007/08 to 2017/18, to derive changes in volume, elevation, thickness, and area.

Explicit volume changes were done using derived glacial thicknesses^20^ and corresponding elevation measurements from DEMs (See Methods). Table 2 presents the summary findings and provides many insights into the recent glaciological history of the region. First it should be noted that all nine glaciers exhibited mass loss during the sixty years. The nine glaciers in total decreased in volume approximately 1.7 km^3^, with an average of 0.2 km^3^ for the nine. The very small Polychrome (0.04 km^3^) changed the least amount (0.01 km^3^) while the large Lemon Creek changed the most (0.56 km^3^). This 1.7 km^3^ of freshwater water equates to 25% of the total glacial volume from the 1958 survey. Thus, 75% of the glacier ice was still remaining as of 2018. The total volume reduction across all nine glaciers (1.7 km³) corresponds to approximately 1.4 metric gigatons of liquid water—enough to submerge the entire city of Los Angeles under 1 m of water.

**Table 2:** Summary of the observed changes in glacial extent over the 60-year observation period: Presented in the table are Volume and Volume Loss from 1957/58 and 2017/18, Liquid Water Lost (in Metric Gt), Area and Area Loss from 1957/58 and 2017/18, and Thickness Loss between 1957/58–2017/18.

Glacier	Volume 1958 (km^3^)	Volume 2018 (km^3^)	Volume loss 1958–2018 (km^3^)	Volume loss percent	Mass of water lost (Gt)	Area 1958 (km^2^)	Area 2018 (km^2^)	Area loss (km^2^)	Area loss percent	Mean thickness loss (m)
Bear Lake	0.88	0.72	0.16	18.40	0.13	6.92	5.41	1.51	21.78	21.63
Blue	0.14	0.08	0.06	41.97	0.05	5.78	5.29	0.49	8.53	9.84
Chikuminuk	0.19	0.11	0.08	43.88	0.07	7.07	6.26	0.80	11.39	10.93
Lemmon creek	2.93	2.37	0.56	19.13	0.46	12.75	9.14	3.61	28.30	40.08
Little jarvis	0.16	0.12	0.04	24.37	0.03	2.48	1.50	0.98	39.56	14.41
McCall	1.00	0.88	0.12	11.62	0.10	7.27	6.37	0.89	12.28	15.89
Polychrome	0.04	0.03	0.01	29.70	0.01	1.85	0.58	1.27	68.63	5.52
West Gulkana	0.25	0.08	0.17	66.66	0.14	4.25	0.57	3.68	86.69	37.77
Worthington	1.22	0.73	0.49	40.16	0.40	9.07	7.32	1.76	19.38	50.32
Total (sum)	**6.81**	**5.12**	**1.69**	**N/a**	**1.39**	**57.44**	**42.44**	**14.99**	**N/a**	**206.39**
Total (mean)	**0.76**	**0.57**	**0.19**	**32.88**	**0.16**	**6.38**	**4.71**	**1.67**	**32.95**	**22.93**

The high-resolution satellite images that support the generation of the DEMs are used in imaging modes to create “pictures” of the glaciers. These pictures were then used in a Geographic Information Systems (See Methods) to perform the area change analysis. In respect to area change, we are interested in both total change in km^2^ as well as percent change which normalizes for small and large glaciers. For example, both West Gulkana and Lemon Creek had a reduction in areal area of approximately 3.6 km^2^ representing an 86% and 28% respective change based on their size. In contrast, Blue had the smallest area change of 0.5 km^2^ resulting in a 9% change over the sixty years. Recall Blue is a northern flowing valley glacier located at a high altitude in the Olympic mountains^21^. In total, the 60 years of extensive mass loss has resulted in 15 km^2^ of the Earth’s surface no longer being glaciated, thus affecting local ecosystems. For example, increased glacial discharge has likely resulted in increased nutrient loading to downstream ecological communities^22^.

The satellite-derived DEM data was also used to obtain elevation change to then estimate loss of glacier thickness. During the observation period, average glacier thickness reduction varied from a minimum of 6 m for Polychrome to a maximum of 50 m for Worthington. The average decrease in thickness for the nine glaciers was approximately 23 m. Notably, our estimated thickness loss for Lemon Creek Glacier (~ 40 m) aligns closely with the ~ 42 m previously reported values reported^23^, providing external validation for the reliability of our approach.

## Discussion

### Volume change scenarios and the survival of the nine

The volume change data was integrated with future atmospheric temperature scenarios to predict percent volume changes relative to 1957 for each of nine glaciers (See Methods). The 1957/58, 1994, 2007/08, and 2017/18 volume data for each of the nine glaciers was used to generate a linear relationship vs. time. Then, the NOAA’s CMIP6 GFDL climate model data^10–12^, which models the past, present, and future air temperatures across the globe under different climate scenarios, was used to infer temperature over time. Specifically, we used the SSP1-2.6^*24*^, SSP2-4.5^*25*^, and SSP5-8.5^*26*^ scenarios corresponding to low, medium, and high emissions, respectively.

**Fig. 3:**
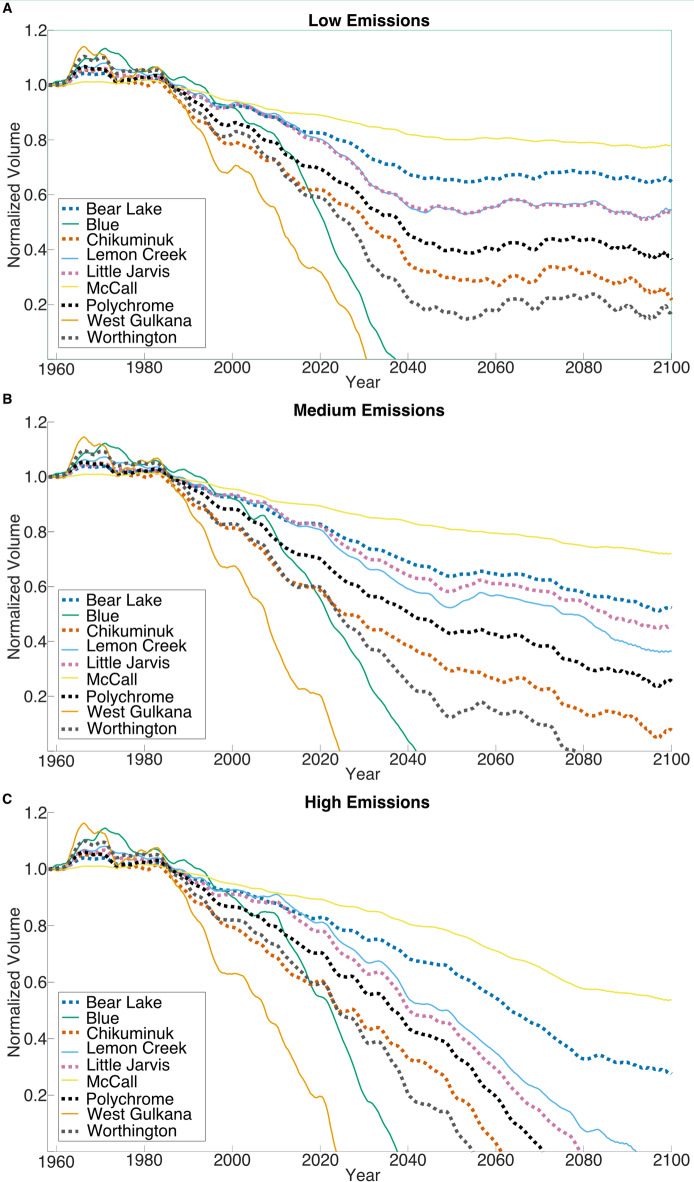
Historical and Future Normalized Volume of the of the nine glaciers: The data in these images reflect the volume change under low (A), medium (B), and high (C) emission scenarios. Notice that by 2100 West Gulkana and Blue Glaciers cease to exist under all three scenarios.

Finally, for each of the nine glaciers, we derived an empirical temperature sensitivity of glacier volume (dV/dT) using the available historical volume observations and corresponding air temperature data. This model was then applied iteratively to year-to-year temperature variations to evolve glacier volume forward in time under low, medium, and high emissions scenarios through 2100 (Fig. 3). The general consensus of the climate community is that unless drastic action is taken, in the year 2100 we will be somewhere between the medium and high emission scenarios. Specifically, the new United Nations climate forecast predicts a mean air temperature increase of 3^o^ C by the year 2100, which falls within the medium scenario^27^.

In the low emission scenario (Fig. 3A) volume at the year 2050 stabilizes to at a smaller volume than at present for seven of the glaciers. Unfortunately, even in the optimistic scenario of small global temperature increase, Blue and West Gulkana have vanished by the year 2035. These two glaciers as indicated in Table 2 are small in area. West Gulkana will cease to exist by 2030. It is a valley glacier^28^ with southern exposure and is subjected to high summer temperatures with low precipitation amounts (46 cm, Table 1). Blue Glacier, which experiences large amounts of perception mostly in the form of rain (540 cm, Table 1) with only slight freezing temperatures in the winter months, will cease to exist by 2040. Blue is a valley glacier in close proximity to the ocean and again is subjected to higher temperatures in the summer months resulting in a ~ 10-meter decrease since 1957/58^29,30^. Note in this low emissions scenario the McCall glacier on the North Slope is the most resilient with only a ~ 20% volume change predicted by the year 2100. McCall is an Alpine Polythermal valley glacier meaning it has a complex thermal structure with cold ice making up the majority of the exposed glacier^31^.

The medium emission scenario (Fig. 3B) indicates that by the year 2100 Blue, West Gulkana and Worthington are totally gone and Chikuminuk’s volume is reduced to less than 10% of its 1957 volume. The other glacier volumes are reduced to values between 25% and 70%. Note for this scenario the McCall glacier, located on the north slope of Alaska, is still the most resilient. Under this medium carbon emissions scenario, it is likely that only five of the nine glaciers will still exist by the year 2100.

In the high emissions scenario (Fig. 3C) we can observe that all but the McCall and Bear Lake glaciers are gone by the year 2100. McCall’s volume would be reduced to approximately 50% while Bear Lake is ~ 30%. Bear Lake is a valley glacier with northwest exposure and is located in a remote part of Alaska and thus less affected by anthropogenic activities. The high emissions climate scenario does not paint an optimistic picture for survival of glaciers throughout North America. McCall is indicative of one of the most resilient types of glaciers (i.e., northernmost/coldest) but yet still loses approximately half of its mass by 2100. Of the nine glaciers, it is the most likely to survive past 2100.

All three climate model runs show an inflection point in glacier volume trends around the year 1990. This inflection reflects a transition in the historical CMIP6 temperature records, not the forward-looking SSP projections, and indicates a shift from relatively stable glacier conditions to more rapid mass loss. Between 1958 and 1990, the glaciers likely experienced intermittent periods of accumulation and comparatively minor losses. However, after 1990, glacier volumes begin declining sharply, coinciding with a documented rise in summer temperatures^32,33^. This timing aligns with previous findings that many of the world’s glaciers entered a period of accelerated negative mass balance around this time^34^. Under the low emissions scenario, glacier volumes begin to stabilize by mid-century, whereas in the high emissions scenario, significant mass loss is projected to continue beyond 2100 for the remaining glaciers.

### Concluding remarks

As part of the 1957/58 IGY, the AGS had the foresight to select nine glaciers that spanned the various types of glaciers that reside in North America. The AGS then in a very labor-intensive manner surveyed these glaciers and created the baseline observations which have been used by investigators to observe change as a result of a warming climate due and anthropogenic forcing. Repeating the initial field measurements today would be cost prohibitive^5^. Fortunately, the onset of many sophisticated high-resolution satellites and the resulting development of satellite produced DEMs has enabled us to do a robust analysis of how these glaciers have changed. This combined past and present detailed dataset then allows for the modeling of their future. Our analysis also validates that high resolution DEMs can be used to monitor glacial changes in remote parts of the world and can be transferred to other glaciers throughout the globe. Given the small uncertainties in the satellite generated DEM’s used in this analysis, the dramatic observed changes in glacial volume are robust. Therefore, the behavior of these glaciers is likely a strong predictor of most glacial behavior in North America.

We have passed the low emissions scenario where seven of the nine glaciers are still standing^35^. The general consensus currently is that we are somewhere between the medium and high emissions scenarios, which recently resulted in the highest ever recorded mean annual summer temperatures (2023/2024)^36^. If current trends continue, we will likely see the death of four to five of the nine glaciers by the year 2100. Since 1958, 25% of the volume of these glaciers has been lost. By 2100, it is likely that 75% of the 1957/58 volume of the glaciers will be gone, showcasing the drastic effect of increasing air temperatures over the next 8 decades. If carbon emissions continue unchecked, it is likely that by the year 2100 a resurvey of these nine glaciers will no longer be necessary.

## Methods

Table S1 provides a concise summary of all elevation datasets used in this study. The table identifies the study period associated with each dataset (historical baseline: 1957/1985; mid-period: 2007/2008; recent: 2017/2018), along with acquisition dates, production methods, and any known limitations or caveats associated with each data source. More detailed descriptions of the generation and processing of the individual elevation products are provided below.

### Historical glacier maps and topography

In 1958, during the International Geophysical Year (IGY), a coordinated federal survey led by the American Geographical Society produced detailed 1:10,000 topographic maps of nine North American glaciers using aerial photography and ground control^4^. These maps featured 5 m contour intervals but were referenced to local coordinate systems, limiting their modern usability.

In 1994, the Geophysical Institute at the University of Alaska Fairbanks resurveyed the glaciers using aircraft-mounted laser altimetry and GPS-based ground profiling^5^. This allowed the IGY maps to be georeferenced to WGS84. This study applied glacier-specific vertical offsets derived from stable bedrock locations to correct systematic biases in the 1958 maps. However, residual uncertainties remain due to limitations in original map quality—particularly in accumulation zones—and the extrapolation of elevation profiles across the glacier surface. Using these corrected and georeferenced maps, we manually digitized glacier outlines and elevation contours in ArcGIS Pro to generate historical DEMs for each glacier’s 1957/58.

### Glacier DEM overview

Within the past 15 years, high-resolution commercial satellite systems have enabled the frequent generation of accurate and high-resolution DEMs in remote regions, including most of the glaciers in this study. To track glacial loss since the 1957/1958 survey, we used surface elevation data for two modern time steps: 2007/2008 and 2017/2018. For Arctic glaciers, DEMs were derived from sub-meter stereo imagery collected by Maxar satellites and accessed through the ArcticDEM database, provided by the Polar Geospatial Center. Imagery was selected mostly during the melt season to reduce snow-related bias and align temporally with the original IGY surveys.

### Intermediate DEMs (2007/2008)

For the 2007–2008 time step, no ArcticDEM products were available for any of the nine glaciers. Glacier surface elevations for this period were therefore derived from high-resolution optical satellite imagery accessed through the U.S. Geological Survey’s National Civil Applications Program (NCAP). These data were processed into imagery-derived products (IDPs) by our late coauthor during his tenure at the USGS.

Digital elevation models were generated using stereo photogrammetric techniques applied to orthorectified imagery, with glacier surfaces manually digitized using consistent ground control and glacier-specific knowledge developed through decades of field and remote-sensing investigations. While detailed procedural documentation for the digitization workflow is no longer available, the resulting DEMs were internally consistent across glaciers and suitable for capturing glacier-scale elevation and volume changes.

These DEMs are treated here as intermediate-quality elevation surfaces and are used to assess decadal-scale change between the 1957–1958 baseline and more recent observations. We acknowledge that these products carry higher uncertainty than modern DEMs derived from contemporary satellite stereo pipelines, and we explicitly account for this limitation as a source of increased uncertainty in mid-period volume change estimates.

### 2017/2018 DEMs

To quantify glacier elevation and volume changes in the most recent decade of the study period, we used two sources of modern elevation data depending on glacier location. For the Arctic glaciers, we used 2-meter resolution ArcticDEM strip products generated from WorldView-1, −2, and − 3 stereo imagery. DEMs were selected from the 2017 and 2018 melt seasons (July–September) to minimize snow cover and cloud contamination, with priority given to strips providing complete glacier coverage and minimal artifacts. All ArcticDEM products were downloaded from the Polar Geospatial Center, orthorectified, and georeferenced to the WGS84 horizontal datum and ellipsoidal vertical datum. DEM strips were visually inspected for artifacts (e.g., voids, steps, blunders). Outliers and spurious values were masked using slope and curvature thresholds, and all elevation data were resampled to a common grid resolution before differencing.

For Blue Glacier, which lies outside the ArcticDEM coverage area, elevation data were obtained from the USGS 3DEP 1/3 arc-second seamless digital elevation model. This product represents a mosaic of the best available elevation data compiled from heterogeneous source datasets rather than a single airborne LiDAR acquisition. Source data contributing to the n48w124 tile were collected between October 2017 and December 2018. Elevations are provided in NAD83 geographic coordinates and referenced vertically to NAVD88. The DEM was resampled to match our standard analysis grid and used to quantify recent glacier change at Blue Glacier.

Glacier outlines for the 2017/2018 period were manually digitized using late summer Sentinel-2 imagery (10 m resolution) accessed through the Copernicus Open Access Hub^18^. These outlines were drawn in ArcGIS Pro and used to constrain the ArcticDEM-derived elevation data. Outlines were based on visible ice boundaries during clear-sky conditions.

### Glacier thickness and bed derivation

Topographic maps and DEMs are valuable for tracking glacial area and surface elevation changes over time, which, when combined with bedrock topography, allow for direct estimates of glacier volume at specific time steps. However, inferring volume change solely from average area and thickness change can be misleading, as doing so implicitly assumes uniform thinning across space. In reality, volume change is better understood as the sum of two components: (1) the volume lost due to complete area retreat, which depends on the average initial thickness over the area lost, and (2) the volume change within the remaining glacier footprint, which depends on observed thinning over that area. Our study avoids this issue by using complete surface and bed elevation data at each time step to calculate total volume, eliminating the need to approximate volume change from area and thickness alone.

To address this, we inferred approximate bed elevation for each glacier by subtracting ice thickness from surface elevation using a recently published global glacier thickness dataset^20^. This dataset estimates glacier thickness for the 2017/2018 period using a mass-conservation approach constrained by satellite-derived surface velocities and DEMs from 2017 to 2018. However, the glacier outlines used in the inversion were sourced from RGI version 6, which, in the Alaska region, generally reflects glacier extents from approximately 2000–2011. As such, while the surface dynamics and elevation reflect modern conditions, some uncertainty may remain in areas where glacier retreat has occurred since the RGI outline dates.

Using the derived bed elevations and available surface DEMs, we calculated glacier volume for each time step and used these values to quantify total volume change over the 60-year study period.

### Climate data

To investigate how historical global and regional temperature trends have affected glacier volumes and to project future changes, we used NOAA’s CMIP6 GFDL climate model^10–12^. This model includes global temperature data from 1921 and projections through 2100 under various climate scenarios. The data, gridded to a 100 km resolution, includes monthly surface temperature averages. For future projections, we used three scenarios: SSP1-2.6^24^, SSP2-4.5^25^, and SSP5-8.5^26^, corresponding to low, medium, and high emissions, respectively.

### Future glacier volume projection

Future glacier volume projections under the three climate scenarios were based on an empirically derived temperature sensitivity of glacier volume (dV/dT). For each glacier, four volume observations (1957/58, 1994/95, 2007/08, and 2017/18) were paired with corresponding annual mean surface air temperatures from the NOAA CMIP6 GFDL climate model to estimate dV/dT.

Following this calculation, glacier volume was then evolved forward in time by iteratively applying this relationship to year-to-year temperature variations. At each time step, glacier volume was updated according to:

$$V\left( {i + 1} \right){\text{ }} = {\text{ }}V\left( i \right){\text{ }} + {\text{ }}\left( {dV/dT} \right){\text{ }} \times {\text{ }}(T\left( {i + 1} \right){\text{ }} - {\text{ }}T(i))$$

such that volume changes accumulate through time in response to the temperature change. Historical and projected temperatures (1958–2100) for each of the three emissions scenarios were used as input to estimate the glacier volume trajectories though the end of the century.

Model performance was evaluated using root mean square error (RMSE) and mean absolute error (MAE) between observed volumes and iteratively modeled volumes at control points. Errors were consistently low across all scenarios (RMSE ≈ 0.028–0.030 km³, MAE ≈ 0.018–0.021 km³), generally corresponding to < 4% of total glacier volume. Projection simulations continued iteratively until modeled glacier volume approached zero or until the year 2100, depending on the temperature trajectory in each scenario.

### Uncertainties

This analysis includes several sources of uncertainty, but the observed glacier changes are substantial enough that these uncertainties likely have minimal impact on the overall results and projections. To start, a glacier ice density of 850 kg/m³ was used to convert volume change to mass^37^. The uncertainty associated with this assumption is small—typically under 10%—and has negligible influence on the interpretation of long-term trends.

The original 1957/1958 mapping uncertainties, documented in the 1998 study, represent a total average error of around 5 m across each map. Although digitization imparts some uncertainty, this is likely minor due to meticulous transcription of points in ArcGIS Pro with sub-meter accuracy. Spatial alignment of DEMs, particularly between historic and modern datasets, represents another potential source of error. While our DEMs were not rigorously co-registered using automated iterative methods, they were carefully inspected at stable, non-glacierized control points and found to have alignment residuals typically under 2–3 m. Vertical offsets were minimized using previously published corrections^5^, and consistent trends across adjacent terrain suggest minimal large-scale warping or tilt. The magnitude of glacier change observed across the study period, often exceeding 40 m in thinning, greatly exceeds any plausible residual georeferencing error. Our estimates for Lemon Creek Glacier, for instance, align closely with previously published values^23^, further supporting the reliability of our approach.

Uncertainties in satellite-derived DEMs are comparatively small, with errors on the order of decimeters^4–6^. This holds true for the mosaiced data for Blue Glacier as well. Ice thickness and bedrock elevation uncertainties average ± 10 m, which influence absolute volume estimates^20^; however, because the same bedrock estimate is applied uniformly across all time periods, these uncertainties do not affect calculated volume change trends.

Observed uncertainty in glacier volume change between 1958 and 2017/18 was quantified by propagating vertical elevation uncertainty into volume units using the fixed integration domain corresponding to the 1958 glacier outline. Vertical uncertainties associated with the historical map-derived DEM and modern DEMs were combined in quadrature and converted to volume uncertainty assuming 1 km² × 1 m = 0.001 km³. Across the nine glaciers, absolute uncertainty in observed volume change ranges from approximately 0.01 to 0.07 km³, while relative uncertainty ranges from roughly 10% to over 90%, reflecting differences in glacier size and total volume loss. Higher percentage uncertainty is associated with small glaciers that experienced limited absolute volume change, whereas larger glaciers exhibit lower relative uncertainty. In all cases, observed volume loss substantially exceeds estimated uncertainty, indicating that the documented glacier changes are robust.

It is also important to acknowledge limitations in glacier boundary delineation from optical remote sensing, particularly in regions of heavy debris cover. In the case of West Gulkana Glacier, a stagnant, debris-covered tongue was excluded from the 2017/18 outline due to a clear disconnection from the active glacier body. This feature lacks dynamic flow or accumulation input and is therefore interpreted as non-active ice, consistent with glacier disconnection definitions^19^. While the omission of such areas may influence modern glacier area estimates, it does not affect volume change calculations, which are based on elevation differencing over a fixed spatial domain (typically the 1958 outline). This methodological approach ensures that inactive regions—whether or not they fall within the most recent glacier outline—are appropriately captured or excluded based on surface elevation trends. Nevertheless, this limitation of optical remote sensing may affect other glaciers in similar ways and introduces a degree of structural uncertainty in late-stage area estimates, particularly where surface debris masks underlying ice.

While climate model uncertainties remain, our use of three forcing scenarios (low, medium, and high emissions) captures a plausible range of future conditions, ensuring robustness in the projection outcomes. Uncertainty in future glacier volume projections was quantified using a Monte Carlo framework that propagates uncertainty in the empirically derived volume–temperature sensitivity (dV/dT) and in the final observed glacier volume. Reported projected volumes correspond to the median and 95% uncertainty interval (2.5–97.5 percentile) of the ensemble. Results of this analysis can be found in Table S2.

For projections, these uncertainties may shift modeled glacier disappearance dates by only a few years, which is minimal on a century scale and does not alter the overall conclusions regarding glacier survival under different climate scenarios.

## Supplementary Information

Below is the link to the electronic supplementary material.

Supplementary Material 1

## Data Availability

Recent glacier DEMs (2017/18) are accessible at https://fridge.pgc.umn.edu/, as well as https://www.sciencebase.gov/catalog/item/4f70aa9fe4b058caae3f8de5 (for Blue). Data is not publicly available for the 2007/2008 period as it is part of the NCAP. Glacier thickness measurements used to derive bed elevations are accessible at https://doi-org.services.lib.mtu.edu/10.6096/1007. Historical glacial DEMs from the 1957/58 season can be obtained by contacting the corresponding author (Dr. Ray Watkins, rhwatkin@mtu.edu) upon request. Climate data (temperature) from each of the scenarios can be obtained from https://cds.climate.copernicus.eu/. Precipitation data can be obtained from https://prism.oregonstate.edu/projects/.
