# Supplementary material for: Sixty years of observations and future projections of nine declining North American glaciers
Source: Sci Rep. 2026 Mar 17;16:13738. doi: 10.1038/s41598-026-41235-6 (PMC13125664; doi:10.1038/s41598-026-41235-6)
Supplement: Supplementary file 1 — Supplementary Material 1 [file 41598_2026_41235_MOESM1_ESM.pdf]

Title: Sixty Years of Observations and Future Projections of Nine Declining North American Glaciers

Supplementary Information

Authors: Edward G. Josberger[1,2] Robert A. Shuchman[2,3], Ray H Watkins[1*]

Affiliations: [1]Michigan Tech Research Institute, Michigan Technological University; Ann Arbor, MI, 48105, USA.

[2]Retired, U.S. Geological Survey, Washington Water Science Center, 1201 Pacific Avenue, Suite 600, Tacoma, WA 98402, U.S.A.

[3]Professor Emeritus, Geological and Mining Engineering and Sciences, Michigan Technological University, Houghton, MI, 49931, USA

*Corresponding author. Email: rhwatkin@mtu.edu

Glacier	Study Period	Data Source	Acquisition Date(s)	Production Method	Known Limitations/ Caveats
**Bear Lake**	Historical baseline	Historical Maps	July, 1957	Aerial Photogroghy/ Modern Digitization	Original Maps did not contain ground control points and were in local coordinate systems, introducing some documented error
	Mid-period	NCAP IDP	2007-2008	Stereo Photogrammetric Techniques	As the data is part of the NCAP IDP program, methods regrading production are not well documented. Additionally, the data is not publicly available
	Recent	Arctic DEM	May, 2018	Commercial Satellite Stereo	Not rigorously co-registered. Potential snowfall may introduce a small positive bias in surface elevations.
**Blue**	Historical baseline	Historical Maps	Sept., 1957	Aerial Photogroghy/ Modern Digitization	Original Maps did not contain ground control points and were in local coordinate systems, introducing some documented error
	Mid-period	NCAP IDP	2007-2008	Stereo Photogrammetric Techniques	As the data is part of the NCAP IDP program, methods regrading production are not well documented. Additionally, the data is not publicly available
	Recent	USGS 3DEP	2017-2018	Mosaicking of multiple data sources	Because the seamless DEM is compiled from source datasets acquired at different times, temporal blending may introduce additional uncertainty in elevation estimates.
**Chikuminuk**	Historical baseline	Historical Maps	July, 1958	Aerial Photogroghy/ Modern Digitization	Original Maps did not contain ground control points and were in local coordinate systems, introducing some documented error
	Mid-period	NCAP IDP	2007-2008	Stereo Photogrammetric Techniques	As the data is part of the NCAP IDP program, methods regrading production are not well documented. Additionally, the data is not publicly available

	Recent	Arctic DEM	June, 2017	Commercial Satellite Stereo	Not rigorously co-registered
**Lemon Creek**	Historical baseline	Historical Maps	Sept., 1957	Aerial Photogroghy/ Modern Digitization	Original Maps did not contain ground control points and were in local coordinate systems, introducing some documented error
	Mid-period	NCAP IDP	2007-2008	Stereo Photogrammetric Techniques	As the data is part of the NCAP IDP program, methods regrading production are not well documented. Additionally, the data is not publicly available
	Recent	Arctic DEM	July, 2017	Commercial Satellite Stereo	Not rigorously co-registered
**Little Jarvis**	Historical baseline	Historical Maps	Sept., 1957	Aerial Photogroghy/ Modern Digitization	Original Maps did not contain ground control points and were in local coordinate systems, introducing some documented error
	Mid-period	NCAP IDP	2007-2008	Stereo Photogrammetric Techniques	As the data is part of the NCAP IDP program, methods regrading production are not well documented. Additionally, the data is not publicly available
	Recent	Arctic DEM	Sept., 2017	Commercial Satellite Stereo	Not rigorously co-registered. Potential snowfall may introduce a small positive bias in surface elevations.
**McCall**	Historical baseline	Historical Maps	Aug., 1958	Aerial Photogroghy/ Modern Digitization	Original Maps did not contain ground control points and were in local coordinate systems, introducing some documented error
	Mid-period	NCAP IDP	2007-2008	Stereo Photogrammetric Techniques	As the data is part of the NCAP IDP program, methods regrading production are not well documented. Additionally, the data is not publicly available
	Recent	Arctic DEM	Aug., 2017	Commercial Satellite Stereo	Not rigorously co-registered

**Polychrome**	Historical baseline	Historical Maps	Aug., 1957	Aerial Photogroghy/ Modern Digitization	Original Maps did not contain ground control points and were in local coordinate systems, introducing some documented error
	Mid-period	NCAP IDP	2007-2008	Stereo Photogrammetric Techniques	As the data is part of the NCAP IDP program, methods regrading production are not well documented. Additionally, the data is not publicly available
	Recent	Arctic DEM	Sept., 2017	Commercial Satellite Stereo	Not rigorously co-registered. Potential snowfall may introduce a small positive bias in surface elevations.
**West Gulkana**	Historical baseline	Historical Maps	July, 1957	Aerial Photogroghy/ Modern Digitization	Original Maps did not contain ground control points and were in local coordinate systems, introducing some documented error
	Mid-period	NCAP IDP	2007-2008	Stereo Photogrammetric Techniques	As the data is part of the NCAP IDP program, methods regrading production are not well documented. Additionally, the data is not publicly available
	Recent	Arctic DEM	Sept., 2017	Commercial Satellite Stereo	Not rigorously co-registered. Potential snowfall may introduce a small positive bias in surface elevations.
**Worthington**	Historical baseline	Historical Maps	July, 1957	Aerial Photogroghy/ Modern Digitization	Original Maps did not contain ground control points and were in local coordinate systems, introducing some documented error
	Mid-period	NCAP IDP	2007-2008	Stereo Photogrammetric Techniques	As the data is part of the NCAP IDP program, methods regrading production are not well documented. Additionally, the data is not publicly available
	Recent	Arctic DEM	May, 2017	Commercial Satellite Stereo	Not rigorously co-registered. Potential snowfall may introduce a small positive bias in surface elevations.

Table S1: Summary of Elevation Sources: All sources used in deriving elevation maps of the glaciers in this study.

Glacier Name	SSP1-2.6 V 2050 (km^3)	SSP1-2.6 V 2100 (km^3)	SSP2-4.5 V 2050 (km^3)	SSP2-4.5 V 2100 (km^3)	SSP5-8.5 V 2050 (km^3)	SSP5-8.5 V 2100 (km^3)
**Bear Lake**	0.57 [0.51 – 0.63]	0.55 [0.49 – 0.62]	0.55 [0.49 – 0.61]	0.45 [0.35 – 0.55]	0.53 [0.50 – 0.62]	0.23 [0.05 – 0.42]
**Blue**	0.00 [0.00 – 0.04]	0.00 [0.00 – 0.03]	0.00 [0.00 – 0.04]	0.00 [0.00 – 0.03]	0.00 [0.00 – 0.03]	0.00 [0.00 – 0.00]
**Chikuminuk**	0.04 [0.00 – 0.10]	0.02 [0.00 – 0.10]	0.04 [0.00 – 0.09]	0.00 [0.00 – 0.08]	0.03 [0.00 – 0.09]	0.00 [0.00 – 0.06]
**Lemon Creek**	1.59 [1.41 – 1.76]	1.55 [1.37 – 1.73]	1.52 [1.35 – 1.70]	1.04 [0.77 – 1.31]	1.42 [1.22 – 1.63]	0.00 [0.00 – 0.35]
**Little Jarvis**	0.08 [0.05 – 0.11]	0.07 [0.04 – 0.11]	0.08 [0.05 – 0.11]	0.06 [0.01 – 0.10]	0.06 [0.03 – 0.10]	0.00 [0.00 – 0.07]
**McCall**	0.79 [0.75 – 0.84]	0.76 [0.70 – 0.82]	0.79 [0.75 – 0.84]	0.70 [0.61 – 0.79]	0.76 [0.70 – 0.82]	0.53 [0.35 – 0.71]
**Polychrome**	0.01 [0.00 – 0.03]	0.01 [0.00 – 0.03]	0.01 [0.00 – 0.03]	0.01 [0.00 – 0.02]	0.01 [0.00 – 0.01]	0.00 [0.00 – 0.01]
**West Gulkana**	0.00 [0.00 – 0.00]	0.00 [0.00 – 0.00]	0.00 [0.00 – 0.00]	0.00 [0.00 – 0.00]	0.00 [0.00 – 0.00]	0.00 [0.00 – 0.00]
**Worthington**	0.19 [0.11 – 0.26]	0.14 [0.05 – 0.22]	0.13 [0.05 – 0.21]	0.00 [0.00 – 0.02]	0.11 [0.02 – 0.20]	0.00 [0.00 – 0.00]

Table S2: Summary of Glacial Change Uncertainty until 2100: Presented in the table are volumes of each glacier in 2050 and 2100 under each climate scenario. The bounds represent the 95% confidence interval for each estimate, propagated from the uncertainty in the glacial topography.
